# Predicting Welding Distortion in a Panel Structure with Longitudinal Stiffeners Using Inherent Deformations Obtained by Inverse Analysis Method

**DOI:** 10.1155/2014/601417

**Published:** 2014-09-03

**Authors:** Wei Liang, Hidekazu Murakawa

**Affiliations:** ^1^College of Mechatronics & Automotive Engineering, Chongqing Jiaotong University, No. 66 Xuefu Road, Nan'an District, Chongqing 400074, China; ^2^State Key Laboratory of Advanced Welding and Joining (AWJ), Harbin Institute of Technology, 92 West Dazhi Street, Nan Gang District, Harbin 150001, China; ^3^Joining and Welding Research Institute, Osaka University, 11-1 Mihogaoka, Ibaraki, Osaka 567-0047, Japan

## Abstract

Welding-induced deformation not only negatively affects dimension accuracy but also degrades the performance of product. If welding deformation can be accurately predicted beforehand, the predictions will be helpful for finding effective methods to improve manufacturing accuracy. Till now, there are two kinds of finite element method (FEM) which can be used to simulate welding deformation. One is the thermal elastic plastic FEM and the other is elastic FEM based on inherent strain theory. The former only can be used to calculate welding deformation for small or medium scale welded structures due to the limitation of computing speed. On the other hand, the latter is an effective method to estimate the total welding distortion for large and complex welded structures even though it neglects the detailed welding process. When the elastic FEM is used to calculate the welding-induced deformation for a large structure, the inherent deformations in each typical joint should be obtained beforehand. In this paper, a new method based on inverse analysis was proposed to obtain the inherent deformations for weld joints. Through introducing the inherent deformations obtained by the proposed method into the elastic FEM based on inherent strain theory, we predicted the welding deformation of a panel structure with two longitudinal stiffeners. In addition, experiments were carried out to verify the simulation results.

## 1. Introduction

Welding-induced deformation not only negatively influences manufacturing accuracy but also degrades the appearance of product. There are too many factors which have influences on the final deformation during welding process, so it is not easy to find a reasonable method to reduce or control welding distortion when only relying on experiments or experiences. However, if welding deformation can be predicted beforehand, the simulation results will be helpful to reduce welding deformation especially in large and complex welded structures. Therefore, it is very important and urgent to develop an effective method to accurately predict welding deformation.

Till present, researchers have proposed two numerical approaches based on FEM to estimate welding deformation. One is thermal elastic plastic FEM [1]. In this method, the welding thermal cycle, the transient stress, the transient strain, and the welding deformation can be simulated when the welding conditions and the temperature-dependent thermal physical and mechanical properties are known. However, because the thermal mechanical behavior is highly nonlinear phenomenon, a very long computing time is required when the thermal elastic plastic FEM is used to calculate welding deformation for large and complex structure. Thus, this method is only suitable for small and medium scale welded structures at present [2].

Because of the limitation of thermal elastic plastic FEM, another method named inherent strain method was proposed [3–5] to effectively predict welding deformation for large and complex welded structures. When the elastic FEM based on inherent strain theory is used to predict welding distortion for a large welded structure, the inherent deformations in each joint should be obtained beforehand. In principle, there are four fundamental types of inherent deformations, namely, longitudinal shrinkage, transverse shrinkage, transverse bending (angular distortion), and longitudinal bending. They are mainly determined by heat input, thickness of plate, and joint type. For a large welded structure, if these four inherent deformation components of each joint are known, the total welding-induced deformation can be predicted using the elastic FEM based on inherent strain theory.

At present, there are two methods which can be used to obtain the inherent deformations for a welded joint. One is the experiment method and the other is thermal elastic plastic FEM. Because the longitudinal shrinkage and longitudinal bending are very small in many cases, it is hard for the experiment method to obtain their accurate values [6, 7]. On the other hand, because the inherent deformations can be expressed by residual plastic strains, the thermal elastic plastic FEM can be used to obtain the components of inherent deformations for a welded joint [8]. In general, when the thermal elastic plastic FEM is used to estimate inherent deformations, the calculation accuracy strongly depends on the thermal physical and mechanical properties, which should be measured by experiment [9]. In addition, the metallurgical factors and heat source models [10] also should be carefully considered in many situations. As mentioned above, there are many factors which can influence the final deformation in the welded structure, so it is not easy to accurately predict welding distortion using thermal elastic plastic FEM, especially for new structural materials because their material properties are very scarce.

To overcome the above disadvantages of both experiment method and thermal elastic plastic FEM, we proposed a new method named inverse analysis method to obtain the inherent deformations for typical joints. This method is a new approach which is based on the combination of experiment and elastic FEM. In the inverse analysis, the coordinates in *x*, *y*, and *z* directions at a small number of locations of a typical joint before and after welding are measured by experiment, and the inherent deformations can be estimated based on these three dimensional (3D) coordinates.

As an example, the inherent deformations of a fillet welded joint were calculated by the proposed method in the present study. Using the estimated inherent deformations, the welding deformation of a panel with two longitudinal stiffeners was predicted by the elastic FEM. Meanwhile, experiments were carried out to verify the simulation results. Through comparing the simulated results and the measurement results, the effectiveness of the developed computational approach based on inverse analysis has been verified.

## 2. Method of Inverse Analysis in Fillet Joint

### 2.1. Hypotheses and Procedure of Inverse Analysis in Fillet Joint

In principle, the welding distortion in a typical joint is mainly caused by four components of inherent deformations, namely, longitudinal shrinkage (*δ*_*xi*_), transverse shrinkage (*δ*_*yi*_), longitudinal bending (*θ*_*xi*_), and transverse bending (*θ*_*yi*_). Another type of deformation is a combination of the above components. According to inverse analysis theory, if the distribution of the inherent deformation is expressed in terms of a small number of parameters, each component of inherent deformation can be determined based on the measured values of deformation at limited locations. In this study, an inverse analysis for obtaining inherent deformation in typical joint was proposed under the following hypotheses.

1. There are four basic components in a typical joint. The four components are longitudinal shrinkage, transverse shrinkage, longitudinal bending, and transverse bending (angular distortion). The first two components are the in-plane deformation and the last two components are out-of-plane deformation.
2. If the distribution function of each component of inherent deformation is expressed by *n* parameters, the total number of parameters is 4*n*.
3. The distribution range (length and width) of inherent deformations in a joint can be determined according to the results obtained by the thermal elastic plastic FEM analysis.
4. The 3D coordinates of *m* points are measured before and after the welding. The joint has no deformation before welding, while it deforms after welding. Through measuring the 3D coordinates before and after welding at limited locations, the basic deformation can be determined.

Since the 3D coordinates measured at *m* points include the rigid body motion, the number of linearly independent relationships is (3*m* − 6). Thus, the necessary condition for determining the inherent deformation is (3*m* − 6) > 4*n*.

**Figure 1:**
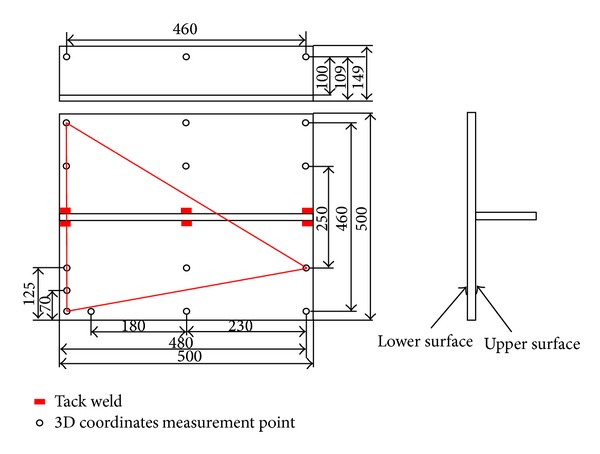
3D coordinate measurement position for inverse analysis.

Based on the above idea, 17 points on the fillet joint as shown in Figure 1 were selected. In the fillet joint, 14 points are selected on both sides of flange and 3 points are selected on web. Through measuring the 3D coordinates at the 17 points, we can determine the inherent deformation *a*_*i*_ (*a*_1_,…, *a*_4*n*_) according to

(1) $$\begin{array}{c} F_{j}\left(a_{i}\right)=F_{j}^{m}, \end{array}$$

where *a*_*i*_ represents the components of inherent deformation and *a* = {*a*_1_,…, *a*_4*n*_}, *F*_*j*_(*a*_*i*_) is the deformation of fillet joint which is computed by the elastic FEM using the inherent deformation *a*_*i*_, and *F*_*k*_^*m*^ is the measured deformation.

**Figure 2:**
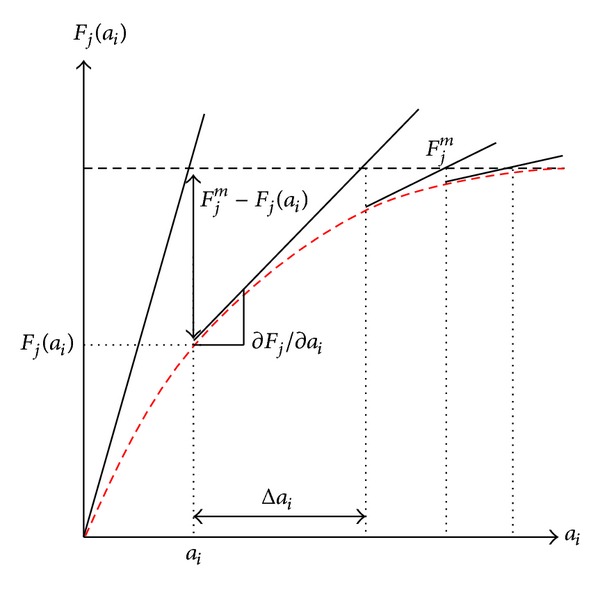
The iterative procedure for obtaining variable *a*_*i*_.

Since the relationship between the inherent deformation *a*_*i*_ and the deformation of the fillet joint *F*_*j*_(*a*_*i*_) is nonlinear, therefore, the inherent deformation *a*_*i*_ cannot be determined from the measured value *F*_*j*_^*m*^ by a single step. It must be determined through an iterative process based on the following Taylor expansion:

(2) $$\begin{array}{c} F_{j}\left(a_{i}+\Delta a_{i}\right)\approx F_{j}\left(a_{i}\right)+\left(\frac{\partial F_{j}}{\partial a_{i}}\right)\Delta a_{i}=F_{j}^{m} \end{array}$$

or in matrix form,

(3) $$\begin{array}{c} \left\{\frac{\partial F_{j}}{\partial a_{i}}\right\}\left\{\Delta a_{i}\right\}=\left\{F_{j}^{m}-F_{j}\left(a_{i}\right)\right\}, \end{array}$$

where Δ*a*_*i*_ is the correction value of the approximate solution at the previous step. *F*_*j*_^*m*^ is the measured deformation. *F*_*j*_(*a*_*i*_) can be computed by the elastic FEM using the inherent deformation *a*_*i*_. The iterative procedure to obtain *a*_*i*_ used in inverse analysis is shown in Figure 2. As seen from the figure, [∂*F*_*j*_/∂*a*_*i*_] is the slope of the curve *F*_*j*_(*a*_*i*_) at a certain point; *a*_*i*_ is calculated by an iterative process until the convergence criterion is met.

The number of rows and columns in matrix [∂*F*_*j*_/∂*a*_*i*_] is 3*m* − 6 and 4*n*, respectively, while the number of rows and columns in matrix {*F*_*j*_^*m*^ − *F*_*j*_(*a*_*i*_)} is 3*m* − 6 and 1, respectively. Thus, Δ*a*_*i*_ should becalculated using the following equation:

(4) $$\begin{array}{c} {\left[\frac{\partial F_{j}}{\partial a_{i}}\right]}^{T}\left[\frac{\partial F_{j}}{\partial a_{i}}\right]\left\{\Delta a_{i}\right\}={\left[\frac{\partial F_{j}}{\partial a_{i}}\right]}^{T}\left\{F_{j}^{m}-F_{j}\left(a_{i}\right)\right\}. \end{array}$$

In addition, to more accurately describe the basic deformation *F*_*j*_^*m*^, a base triangle which consists of points (1), (2), and (3) as shown in Figure 1 was defined. The 3*m* − 6 variables in the arrays *F*_*j*_^*m*^ are defined as follows.

1. From the coordinates at the three points belonging to the base triangle, the length change of each side after welding can be calculated, and, hence, the three variables can be obtained.
2. Concerning the other 15 points, the variations of the distances after welding from each point to arbitrary two points belonging to the basic triangle can provide 2(*m* − 3) variables. In addition, from the variations of the normal distance from each point to the plane determined by the base triangle, (*m* − 3) variables can be obtained.
3. Form (2) and (3), (3*m* − 6) variables can be obtained.

### 2.2. Finite Element Model of Inverse Analysis in Fillet Joint

**Figure 3:**
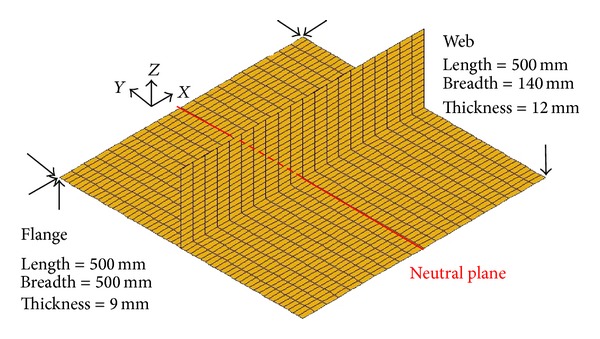
FEM mesh and restraint conditions in fillet joint.

In the developed elastic FEM, four-node plate elements (shell element) are used to simulate welding deformation. The dimensions of the finite element model, the mesh division, and the boundary conditions of the model are shown in Figure 3. In this model, the length of both the flange and the web is 500 mm, the breadth of the flange is 500 mm, and the height of the web is 150 mm. The thickness of the flange is 9 mm and that of the web is 12 mm, respectively. The fillet joint was welded without any external restraint during the entire experiment process, so the boundary conditions were just used to prevent rigid body motion on the mechanical analysis. The elastic strain was modeled using the isotropic Hook's law with Young's modulus and Poisson's ratio at room temperature.

Using the center coordinates of the 17 holes measured before and after welding, the four components of the inherent deformations in fillet joint can be estimated by inverse analysis.

### 2.3. Experimental Procedure

In order to obtain the inherent deformations in a fillet joint, an experimental mock-up was built up to measure the 3D coordinates at 17 locations in the fillet joint before and after welding. The welding was performed and the measuring was carried out according to the following procedure.

**Figure 4:**
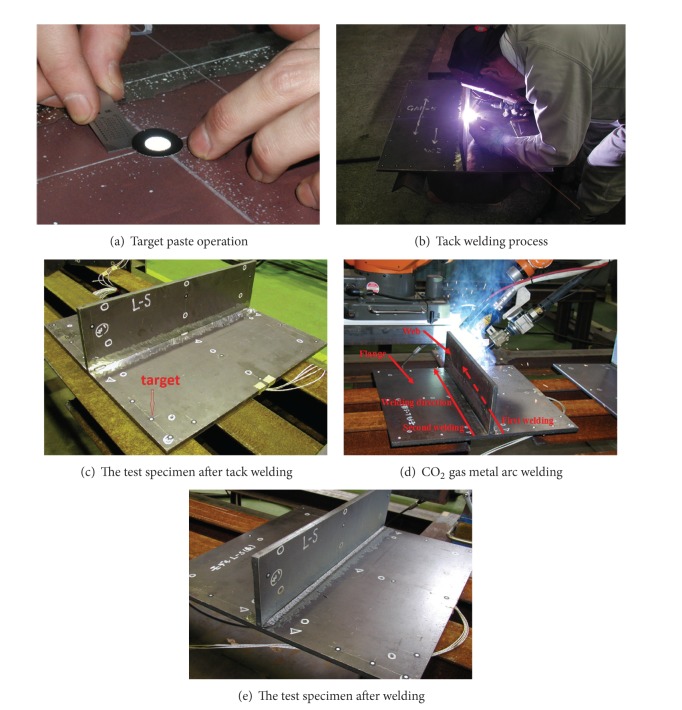
Experimental procedure.

1. In the experiments, a 3D photograph technique was used to measure the 3D coordinates in limited locations. After the experimental mock-up was prepared well, the targets were pasted on the surface of the mock-up. The locations of these targets are shown in Figure 1. The picture of target-pasting operation is shown in Figure 4(a).
2. Before welding, the web and the flange were tack-welded at first. The locations of tack weld are shown in Figure 1 by the short bars. The length of each tack weld was approximately 10 mm. The tack welds were performed by TIG welding process. The pictures of tack welding process apparatus and the test specimen after tack welding are shown in Figures 4(b) and 4(c). In Figure 4(c), the white spots are the targets.
3. The long welding lines were performed by CO_2_ gas metal arc welding using a single-sided welding procedure. The welding current, the arc voltage, and the welding speed were 270 A, 29 V, and 400 mm/min, respectively. The angle of torch was 45 degrees. The filler metal was flux-cored wire, whose chemical composites are almost the same as those of the base material. The pictures of welding apparatus and the test specimen after welding are shown in Figures 4(d) and 4(e). Welding direction is shown by the two solid arrows in Figure 4(d). The dimensions of experimental specimen are the same as those of the finite element model as shown in Figure 3. The material used in this study was shipbuilding steel SM400A.

A digital camera was employed to record the 3D coordinates of each target before and after welding [11]. Using these 3D coordinate data, the welding distortion can be calculated by using the developed inverse analysis approach based on elastic FEM.

### 2.4. Computed Results and Discussion

Using the coordinates of the 17 points measured before and after welding, the four components of the inherent deformations can be estimated by the inverse analysis described above. For the fillet joint, because the distortion of web was very small, in this work, the inherent deformations were assumed to be equivalently distributed in the left and right sides of flange near the welding line.

**Figure 5:**
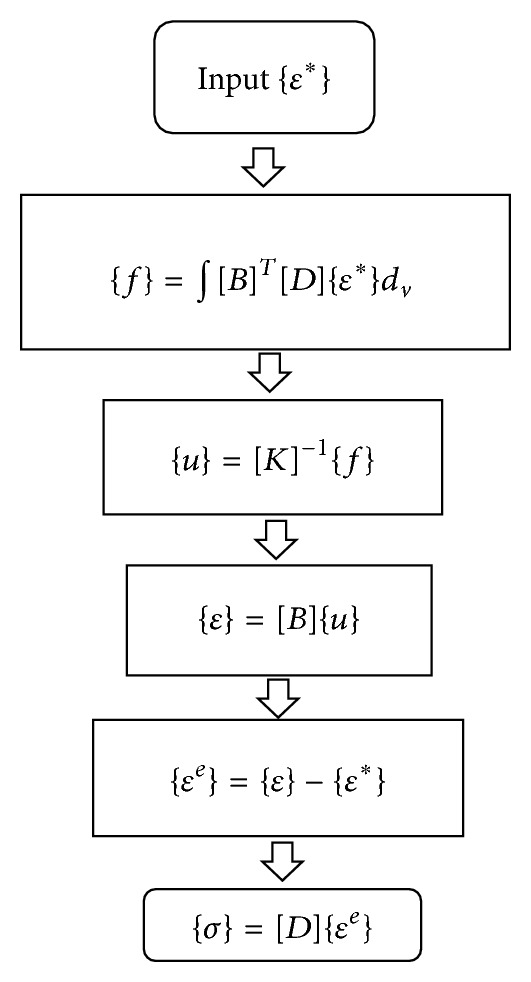
Analysis procedure of inherent strain method.

As the result of inverse analysis, each component of inherent deformations, namely, longitudinal shrinkage, transverse shrinkage, longitudinal bending, and transverse bending (angular distortion), is summarized in Table 1. To examine the effectiveness of the estimated inherent deformations, the welding deformation produced by these inherent deformations was computed using a forward analysis method. The forward analysis procedure is shown in Figure 5. In Figure 5, {ε*},  {*ε*},  {*ε* ^*e*^},  {*f*},  {*u*}, and {*σ*} are vectors of inherent strain, total strain, elastic strain, equivalent nodal load, nodal displacement, and residual stress, respectively. [*B*], [*D*], and [*K*] are strain-displacement matrix, elastic stress-strain matrix, and stiffness matrix, respectively. Using the inherent deformations shown in Table 1, the welding deformation of fillet model was simulated by the elastic FEM.

**Figure 6:**
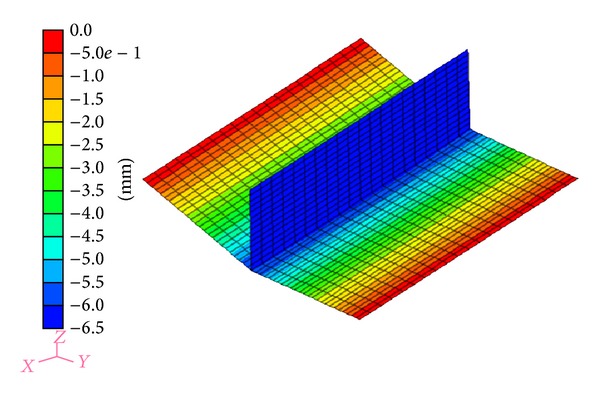
*Z*-displacement of fillet joint model calculated by elastic FEM.

In this study, angular distortion and transverse shrinkage are investigated by means of experiment and numerical simulation. As an example, the distribution of the displacement in *z*-direction (angular distortion) is shown in Figure 6. From this figure, it can be seen that the maximum deflection which is predicted by inverse analysis is 6.5 mm. The angular distortion is significant.

**Figure 7:**
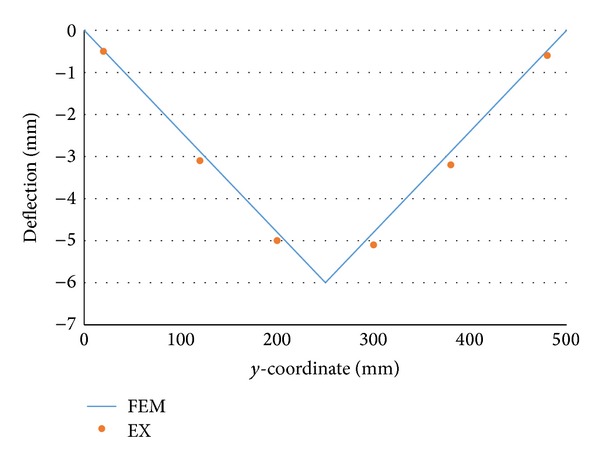
Deflection distribution in the neutral plane.

To clarify if the average values of each component of inherent deformations can be used to accurately predict welding deformation, the deflection along the neutral plane is plotted in Figure 7 using blue line. In the figure, the measured values are represented by solid circles. As seen from Figure 7, the predicted deformations using the average values of each inherent deformation obtained by inverse analysis method are close to the experimental measurements values.

## 3. Prediction of Welding Deformation in Panel Structures with Longitudinal Stiffeners

### 3.1. Experimental Model

**Figure 8:**
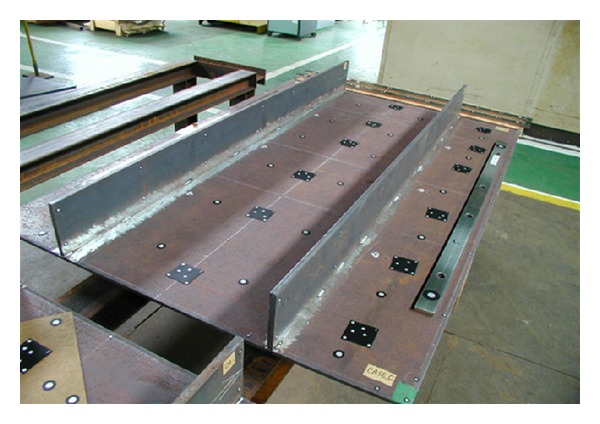
Experimental model of panel structures.

**Figure 9:**
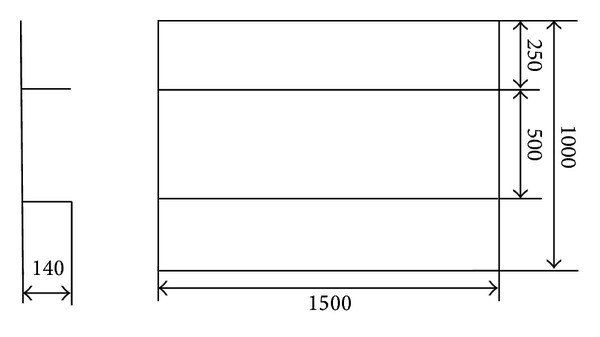
Dimensions of the panel structure.

In order to investigate the calculated precision of welding deformation for relatively large structure using the inherent deformations obtained by the proposed inverse analysis, one experimental mock-up as shown in Figure 8 was manufactured. The mock-up consists of a skin plate and two longitudinal stiffeners.The dimensions of the mock-up are shown in Figure 9. The thickness of the skin is 9 mm and that of the longitudinal stiffeners is 12 mm, respectively, which is the same as the fillet joint described in Section 2.2.

In the experiments, in order to avoid the influence of welding sequence on the final distortion, all members in the welded structures were tack-welded before welding. The length of each tack weld bead is approximately 15 mm. The experimental material, the welding methods, and the welding condition are the same as those of the experimental model as mentioned in Section 2.3.

In the experiments, 3D photography technique was also used to measure welding distortion. The detailed description can be found in [11]. After the experimental model was prepared well, targets were pasted on the surface. The arrangement of these targets is shown in Figure 8. In this figure, the white spots are target. A digital camera was employed to record the coordinates of each target. Using the 3D coordinate data before and after welding, the welding distortion can be calculated.

### 3.2. Brief Introduction of Computational Approach

**Figure 10:**
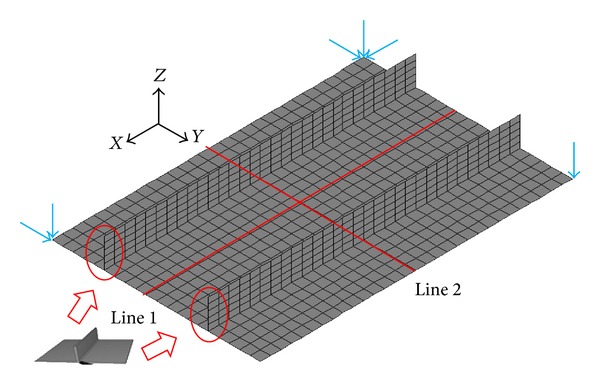
FEM model of the panel structure.

The welding deformation of the panel structure, whose dimensions are the same as those of the mock-up, was predicted by elastic FEM based on inherent strain theory. The mesh of the model is shown in Figure 10. In the panel structure, because the plate thickness of the web as well as the flange is identical to that of the fillet joint and the welding conditions are also the same as those used in the fillet joint as described in Section 2.3, we can introduce the inherent deformations of the fillet joint into the panel model as the initial strains.

### 3.3. Simulation Results

**Figure 11:**
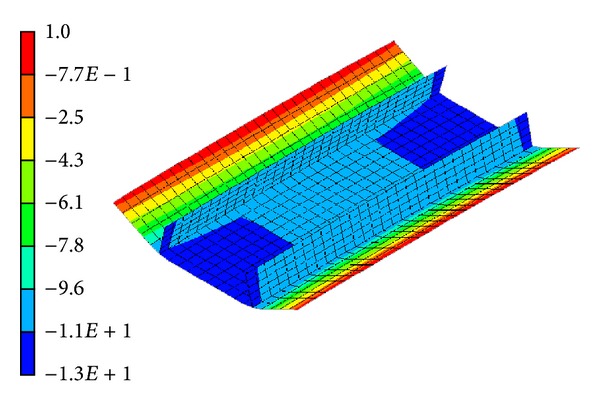
*Z*-displacement distribution.

**Figure 12:**
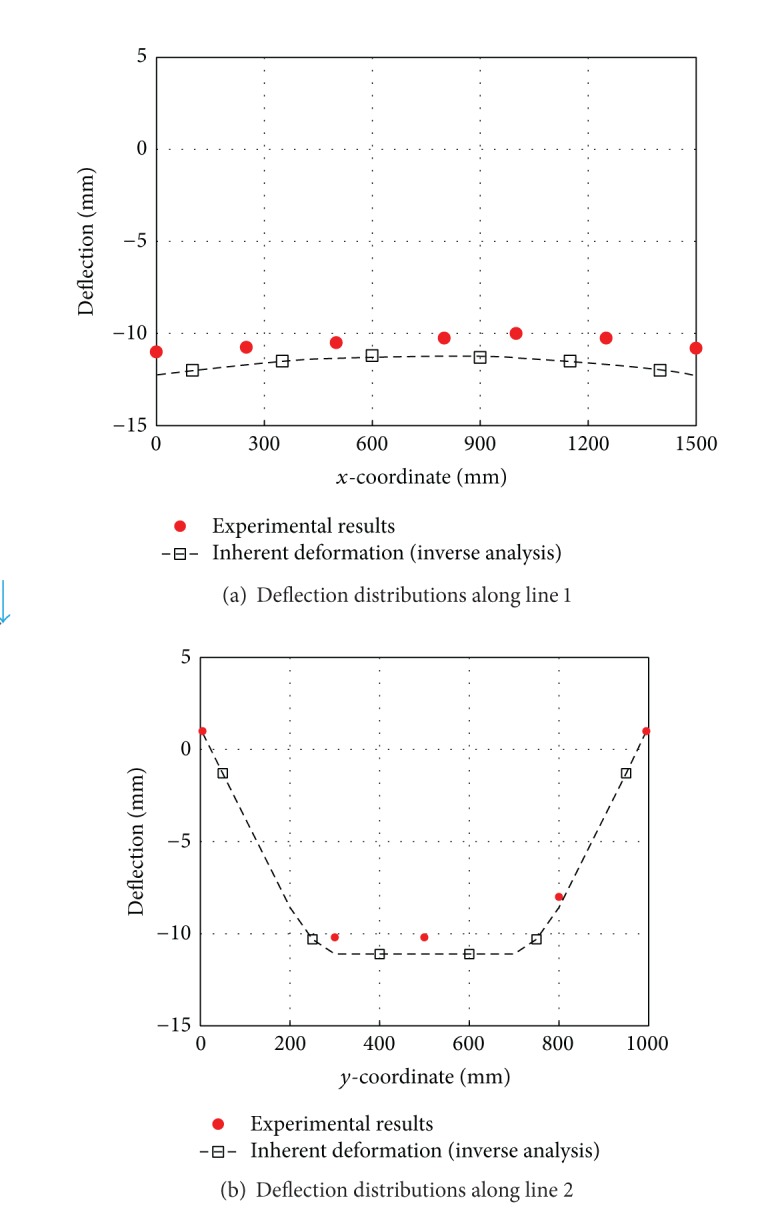
Comparison of deflection between experiments and prediction.

The final deflection of panel model is shown in Figure 11. This figure tells us that the difference between the maximum deflection and the minimum deflection is about 14 mm. To compare the simulated results and the measured data, we selected two compared paths (line 1 and line 2) as shown in Figure 10 to plot their deflection distributions. Figures 12(a) and 12(b) compare the deflection distributions along line 1 and line 2, respectively. In these two figures, the measured data are represented by solid spots and the deflections predicted by the elastic FEM results are represented by black line. As seen from the figures, the predictions are in a good agreement with the experimental measurements. Moreover, the simulated results have proved that the inherent deformations estimated by inverse analysis can reasonably and effectively predict the welding distortion for the panel structure with two longitudinal stiffeners. Carefully observing Figure 12, we can find that the simulated deflection has a symmetric distribution, while the measured deflection has an asymmetric distribution. In the simulation, it was assumed that the inherent deformations are uniform along the welding line, so it is natural that the final deflection distribution along welding is symmetric. On the other hand, because the welding arc moved along the welding line with a certain speed, it inevitably resulted in an asymmetric deflection distribution. Even though there is a difference between the predictions and measurements, the discrepancy is not notable. On the whole, the simulated results obtained from the elastic FEM based on the inherent strain theory match the measured data well. This suggests that the inverse analysis method is a useful and effective way to obtain inherent deformations for fillet joint.

## 4. Conclusions

In this work, a new method based on inverse analysis was proposed to obtain the inherent deformations for a fillet joint. Using the estimated inherent deformations, the welding deformation of a panel structure with two longitudinal stiffeners was simulated. Through comparing the prediction and the measurements results, the following conclusions can be drawn.

1. Using the 3D coordinates of the 17 points measured before and after welding, the four components of the inherent deformations in a fillet joint can be estimated by the inverse analysis.
2. Using the average value of the four components of inherent deformations along welding line obtained by inverse analysis, the welding distortion of fillet joint was simulated by the FEM based on inherent strain theory. The good agreement between the predictions and the measured data demonstrates that the average value of each inherent deformation component along the welding line estimated by the proposed inverse analysis can be regarded as equivalent value.
3. Using the inherent deformations of the fillet joint, the welding deformation in a panel structure with two longitudinal stiffeners was predicted. Through comparing the simulated result and the measured data, we found that the inherent deformations estimated by the inverse analysis can accurately predict the total welding deformation for the panel structure. The elastic FEM analysis can be completed in a very short time and the short computing time is very meaningful for practicing engineering analysis.

## Figures and Tables

**Table 1:** Inherent deformations in the fillet jount.

Method	Longitudinal shrinkage (mm)	Transverse shrinkage (mm)	Longitudinal bending (rad)	Transverse bending (angular distortion) (rad)
Inverse analysis	−0.1	−0.22	0.0003	0.024
